# Treatment of hyperprolactinemia in women: A Position Statement from the Brazilian Federation of Gynecology and Obstetrics Associations (Febrasgo) and the Brazilian Society of Endocrinology and Metabolism (SBEM)

**DOI:** 10.20945/2359-4292-2023-0504

**Published:** 2024-04-09

**Authors:** Cristina Laguna Benetti-Pinto, Andrea Prestes Nácul, Ana Carolina Japur de Sá Rosa-e-Silva, Gustavo Arantes Rosa Maciel, Vania dos Santos Nunes Nogueira, Paula Condé Lamparelli Elias, Manoel Martins, Leandro Kasuki, Heraldo Mendes Garmes, Andrea Glezer

**Affiliations:** 1 Universidade Estadual de Campinas Faculdade de Ciências Médicas Departamento de Obstetrícia e Ginecologia Campinas SP Brasil Departamento de Obstetrícia e Ginecologia, Faculdade de Ciências Médicas, Universidade Estadual de Campinas, Campinas, SP, Brasil; 2 Unidade de Reprodução Humana Hospital Fêmina Grupo Hospitalar Conceição Porto Alegre RS Brasil Unidade de Reprodução Humana, Hospital Fêmina, Grupo Hospitalar Conceição, Porto Alegre, RS, Brasil; 3 Universidade de São Paulo Faculdade de Medicina de Ribeirão Preto Departamento de Ginecologia e Obstetrícia Ribeirão Preto SP Brasil Departamento de Ginecologia e Obstetrícia, Faculdade de Medicina de Ribeirão Preto, Universidade de São Paulo, Ribeirão Preto, SP, Brasil; 4 Universidade de São Paulo Faculdade de Medicina Hospital das Clínicas São Paulo SP Brasil Departamento de Obstetrícia e Ginecologia, Disciplina de Ginecologia, Hospital das Clínicas, Faculdade de Medicina, Universidade de São Paulo, São Paulo, SP, Brasil; 5 Universidade Estadual Paulista Faculdade de Medicina de Botucatu Departamento de Clínica Médica Botucatu SP Brasil Departamento de Clínica Médica, Faculdade de Medicina de Botucatu, Universidade Estadual Paulista, Botucatu, SP, Brasil; 6 Universidade de São Paulo Faculdade de Medicina de Ribeirão Preto Hospital das Clínicas Ribeirão Preto SP Brasil Departamento de Clínica Médica, Hospital das Clínicas, Faculdade de Medicina de Ribeirão Preto, Universidade de São Paulo, Ribeirão Preto, SP, Brasil; 7 Universidade Federal do Ceará Núcleo de Pesquisa e Desenvolvimento de Medicamentos Departamento de Medicina Clínica Fortaleza CE Brasil Departamento de Medicina Clínica e Núcleo de Pesquisa e Desenvolvimento de Medicamentos, Universidade Federal do Ceará, Fortaleza, CE, Brasil; 8 Universidade Federal do Rio de Janeiro Hospital Universitário Clementino Fraga Filho Rio de Janeiro RJ Brasil Hospital Universitário Clementino Fraga Filho, Universidade Federal do Rio de Janeiro, Rio de Janeiro, RJ, Brasil; 8 Universidade Estadual de Campinas Faculdade de Ciências Médicas Campinas SP Brasil Faculdade de Ciências Médicas, Universidade Estadual de Campinas, Campinas, SP, Brasil; 10 Universidade de São Paulo Faculdade de Medicina Hospital das Clínicas São Paulo SP Brasil Hospital das Clínicas, Faculdade de Medicina, Universidade de São Paulo, São Paulo, SP, Brasil

**Keywords:** Prolactinoma, prolactin, dopamine agonist, cabergoline, pregnancy

## Abstract

Dopamine agonists are the first line of treatment for patients with symptomatic hyperprolactinemia due to prolactinomas and in those with idiopathic hyperprolactinemia. Treatment with these agents is effective in 80%-90% of the cases. Infertility treatment of patients with hyperprolactinemia is also carried out with dopamine agonists, aiming for the normalization of prolactin levels. The risk of symptomatic growth of prolactinomas during pregnancy is dependent on the tumor's size, duration of previous treatments, and prolactin levels. Notably, the corresponding risk is relatively low in cases of microprolactinomas (<5%). Remission of hyperprolactinemia occurs in about 30% of the patients after drug treatment and may also occur after pregnancy and menopause. The use of some drugs, such as antidepressants and antipsychotics, is a frequent cause of hyperprolactinemia, and managing this occurrence involves unique considerations. This position statement by the Brazilian Federation of Gynecology and Obstetrics Associations (Febrasgo) and Brazilian Society of Endocrinology and Metabolism (SBEM) addresses the recommendations for measurement of serum prolactin levels and the investigations of symptomatic and asymptomatic hyperprolactinemia and drug-induced hyperprolactinemia in women.

## KEY POINTS

- Hyperprolactinemia is a cause of menstrual irregularity, galactorrhea, hypogonadism, and infertility. Recognizing the cause of hyperprolactinemia is essential for the implementation of appropriate treatment.
- Drug treatment with dopamine agonists is the first-line therapy for idiopathic hyperprolactinemia or symptomatic hyperprolactinemia due to prolactinoma.
- Treatment with dopamine agonists is effective in 80%-90% of the cases.
- Infertility treatment in patients with hyperprolactinemia is carried out with dopamine agonists, aiming for the normalization of prolactin levels.
- The risk of symptomatic growth of prolactinomas during pregnancy is related to the size of the tumor, duration of previous treatment, and higher prolactin levels. The risk of growth of microprolactinomas during pregnancy is below 5%.
- Remission of hyperprolactinemia can occur after pregnancy and menopause. Around 30% of the patients experience remission after treatment with dopamine agonists.
- Antidepressants and antipsychotics are a frequent cause of hyperprolactinemia, a situation that requires specific management.

## RECOMMENDATIONS

- The initial treatment of patients with hyperprolactinemia caused by prolactinomas or idiopathic hyperprolactinemia is pharmacological, and dopamine agonists are the medications of choice. Cabergoline is more effective and better tolerated than bromocriptine and is the first therapeutic choice.
- Treatment should begin with lower doses and gradual dose progression for better tolerability. The use of high doses requires attention to potential side effects and psychiatric effects, as well as monitoring for valvular heart disease.
- In cases of optimal control of symptoms, normalized prolactin levels, and effective management of prolactinomas with dopamine agonists, treatment for a minimum of 2 years is recommended before dose reduction or medication withdrawal.
- In symptomatic patients with prolactinomas, surgical treatment may be indicated in cases of intolerance or resistance to dopamine agonists.
- Symptomatic growth of microadenomas is infrequent during pregnancy. Dopamine agonists can be withdrawn during pregnancy in cases of idiopathic hyperprolactinemia, microadenomas, and intrasellar macroadenomas that show optimal tumor control after at least 1 year of drug treatment before pregnancy.
- Tumors diagnosed during reproductive years and showing optimal control with dopamine agonists may have treatment discontinued in menopause.
- The status of the prolactinoma must be reevaluated with pituitary imaging after 1-2 years of treatment with a dopamine agonist and after pregnancy or menopause. Exceptions to this rule are macroadenomas with a risk of compression or inadequate response, which must be individually reevaluated.
- The patient should be referred to a specialist in cases of hypopituitarism or hyperprolactinemia due to stalk disconnection, acromegaly, or resistant and aggressive prolactinomas.
- Patients with hyperprolactinemia due to medications should be referred to the prescribing physician, who should decide whether to suspend or replace the medication. If the suspension or replacement of the medication is unfeasible, pituitary imaging should be performed to rule out the presence of a tumor, and hormone replacement containing estrogen should be considered in the presence of hypogonadism to reduce risks.

## BACKGROUND

The preferred treatment for hyperprolactinemia, when deemed necessary, involves the administration of a dopamine agonist over an extended period. In the context of hyperprolactinemia, the female body has unique characteristics, as the menstrual cycle, menopause, and pregnancy influence prolactin secretion. Estrogen, the main ovarian hormone, influences prolactin secretion through different mechanisms, *i.e.*, regulation of prolactin gene expression, downregulation of dopamine receptor expression, and stimulation of lactotroph proliferation. Due to these effects, estrogen is considered a prolactin-releasing factor. During pregnancy, circulating estrogen levels are high and may stimulate the secretion of prolactin and the growth of prolactinomas. In contrast, the reduction in ovarian estrogen secretion during menopause reduces the stimulation of estrogen on prolactin secretion and the proliferation of lactotrophs. Notably, prolactin is the only pituitary hormone regulated primarily through hypothalamic tonic inhibition (via dopamine). Therefore, interrupting the transport of dopamine from the hypothalamus to the pituitary by compression or inflammation of the pituitary stalk or effects of different medications may result in hyperprolactinemia. In women with confirmed hyperprolactinemia, therapeutic decisions are influenced by both the etiology of the hyperprolactinemia and the woman's reproductive status.

### When should treatment for hyperprolactinemia be initiated?

The treatment of hyperprolactinemia with dopamine agonists is recommended for symptom control and, in cases of macroprolactinomas, for the effects of these agents on the tumor mass (1).

Thus, treatment indications for hyperprolactinemia include:

- Control of symptoms secondary to dysfunction of the hypothalamic-pituitary-gonadal axis, including menstrual irregularity or amenorrhea, infertility, and decreased libido.
- Bothersome galactorrhea.
- Desire for conception.
- Neurological symptoms.
- Control of tumor mass (in cases of macroprolactinomas).

### When should hyperprolactinemia not be treated?

In cases of microprolactinomas in asymptomatic patients, treatment may be discussed and the patients can be monitored without therapy (1). In patients with amenorrhea secondary to a microprolactinoma who do not wish to become pregnant, combined hormonal contraceptives may be an alternative to dopamine agonists (1,2). Patients for whom follow-up or use of oral combined hormonal contraceptives is chosen must receive proper guidance and be involved in the discussion about the importance of regular monitoring with tumor imaging and the possibility of introducing a dopamine agonist in case of tumor growth. In cases of hyperprolactinemia secondary to hypothyroidism, the thyroid dysfunction must be treated first, and once the thyroid function is normal, hyperprolactinemia-related symptoms and serum prolactin levels should be reevaluated. The discussion of hyperprolactinemia secondary to medications will be addressed in a later section.

### WhenHow should hyperprolactinemia be treated and for how long?

Dopamine agonists are considered the first-line treatment for prolactinomas and idiopathic hyperprolactinemia (1,2). These agents act by binding to dopamine D2 receptors, which are highly expressed in prolactinomas (3). Two dopamine agonists are available in Brazil, i.e., bromocriptine and cabergoline. These agents normalize serum prolactin levels and reduce the size of prolactinomas in most cases. Cabergoline is considered the drug of choice due to greater efficacy (normalization of prolactin levels in 95% of the cases, compared with 80% with bromocriptine) and better dosage and tolerability. The main characteristics of bromocriptine and cabergoline are shown in Table 1.

**Table 1:** Main characteristics of the two dopamine agonists commercially available in Brazil

	Cabergoline	Bromocriptine
Recommended average doses	0.5-2 mg	2.5-7.5 mg
Biochemical efficacy	Up to 95%	Up to 80%
Half-life	63-69 hours	6-20 hours
Usual dosage	Once to twice a week	Two to three times a day

Source: Adapted from Shibli-Rahhal & Schlechte (2011) (4).

Treatment with cabergoline starts at a dose of 0.25-0.5 mg once a week in cases of microprolactinomas and idiopathic hyperprolactinemia and 0.5 mg twice a week in most patients with macroprolactinomas. Prolactin levels should be measured every 4-8 weeks during treatment, and the cabergoline dose should be increased progressively if prolactin does not normalize (1,2). In patients with microprolactinomas who achieve normal prolactin levels with cabergoline, pituitary magnetic resonance imaging (MRI) can be repeated after 1-2 years of treatment. For most patients with macroprolactinomas without compressive symptoms and with good response to dopamine agonists, reevaluation with pituitary MRI after 6 months of treatment is appropriate, but in cases with visual loss, the MRI should be repeated 1-3 months after treatment initiation (1,2). A neuro-ophthalmologic evaluation should be performed in patients with macroprolactinomas abutting optical pathways and must be repeated in cases with visual impairment (1). Remission of prolactinomas with dopamine agonists may occur and is mainly achieved after 2 years of treatment (5). Indeed, a recent meta-analysis showed that 36.6% of patients with hyperprolactinemia who discontinued dopamine agonists remained in remission; the remission rate was even higher in patients treated with cabergoline (41.2%) (6). Progressive dose reduction and withdrawal of dopamine agonists may be considered after 2 years of treatment in asymptomatic women with normal prolactin levels and no residual tumor or with important tumor reduction on MRI. This is particularly applicable to tumors without signs of invasion into the cavernous sinus and located at a safe distance from the optic pathways. The risk of relapse is greatest in the first year after treatment suspension. Therefore, serum prolactin levels should be measured every 3 months in the first year post-treatment and at longer intervals thereafter (1,2). If hyperprolactinemia recurs after treatment suspension, a second attempt to withdraw the dopamine agonist may be undertaken after 2 more years of treatment, provided that normal prolactin levels are maintained (7).

### What are the challenges related to the treatment with dopamine agonists?

The main challenges related to the use of dopamine agonists are intolerance and resistance to these agents.

### What are the mechanisms and frequency of intolerance to dopamine agonists and the most common adverse events?

The most common adverse events of dopamine agonists are related to their action on dopamine receptors and include dizziness, postural hypotension, nausea, vomiting, headache, constipation, and gastroesophageal reflux (Table 2) (8). Most of these adverse events are dose-dependent.

**Table 2:** Adverse effects of dopamine agonists

Most common adverse effects	Nausea
	Headache
	Dizziness, postural hypotension
	Abdominal pain, dyspepsia
	Fatigue
	Nasal congestion
Occasional adverse effects	Vomiting
	Constipation
	Leg cramps
Rare adverse effects	Insomnia
	Raynaud's phenomenon
	Heat waves
	Thromboembolic phenomena*
	Valvular heart disease
	Depression, anxiety, psychosis
	Pleural/pulmonary fibrosis
	Constrictive pericarditis
	Dyskinesia
	Paresthesia
	Psychiatric events (compulsive behavior)

* No causal relationship has been demonstrated to date.

Source: Adapted from Olafsdottir & Schlechte (2006) (9).

Compared with bromocriptine, cabergoline demonstrates a comparable safety profile, with adverse effects typically manifesting less frequently (Table 3) and being less severe and of shorter duration; these effects often resolve with dose reduction or continuous use, which is notably reflected in discontinuation rates, where bromocriptine exhibits a 12% rate and cabergoline a 3% rate (10,11).

**Table 3:** Adverse effects of bromocriptine and cabergoline treatment in women with hyperprolactinemia

		Bromocriptine (%) n = 251	Cabergoline (%) n = 221
Gastrointestinal tract	Nausea	50	31
	Vomiting	10	4
	Constipation	9	7
	Dry mouth, dyspepsia, reflux	Rare	N/A
Cardiovascular system	Postural hypotension	26	25
	Flushing, nasal congestion	Rare	N/A
Neurological system	Headache	29	30
	Psychosis, mania, paresthesia, nightmares, blurred vision	Rare, high doses	N/A

Abbreviation: N/A, not applicable.

Source: dos Santos Nunes et al. (2011) (10) and Webster et al. (1994) (11).

### How to prevent or minimize the most common adverse effects of dopamine agonists?

Low doses of dopamine agonists followed by a gradual increase are recommended at treatment start, with the medication taken after meals (12). Serotonin receptor antagonists acting as antiemetics (*e.g.*, ondansetron) should not be taken together with dopamine agonists, as they may increase the hypotensive effect of the latter.

### Valvular heart diseases

The cardiac safety of dopamine agonists has been questioned after these drugs were associated with valvulopathy in patients with Parkinson's disease (13). The pathophysiology involved in this complication relates to the proliferation of fibroblasts under the valves and chordae tendineae by an agonistic action on the 5-HT2B serotonin receptor. Cabergoline acts as an agonist, while bromocriptine functions as a partial agonist for this receptor. Patients with Parkinson's disease are generally older, have more comorbidities, and receive much higher doses of cabergoline than those with hyperprolactinemia. In Brazil, the present package insert for cabergoline advises conducting an echocardiographic evaluation before initiating the medication. In other countries, like the United States, periodic echocardiographic evaluation is recommended during treatment with cabergoline. Several studies have been published since 2008 on the cardiac effects of dopamine agonists. Most of these studies show that the commonly used doses (cabergoline up to 2.0 mg weekly) are not associated with clinically significant valvular heart disease. However, a 2018 meta-analysis of 13 studies assessing the treatment of hyperprolactinemia with cabergoline for at least 6 months found an increased risk of mild or clinically significant (moderate or severe) tricuspid insufficiency (14).

### Considering costs and benefits, who should be evaluated with echocardiography?

When dopamine agonists are started for hyperprolactinemia treatment, a clinical assessment of the cardiac risk is recommended. The assessment can be conducted through medical history and physical examination. In patients at higher cardiac risk, at clinical discretion, echocardiographic evaluation can be obtained where available. Cabergoline should be avoided in patients with moderate to severe valvular abnormalities manifesting clinically. For most patients with optimal control who can tolerate dopamine agonists at standard doses, the risk of developing valvular heart disease appears to be very low. In patients with resistant prolactinomas, *i.e.*, those who require higher cabergoline doses (>2.0 mg weekly), we suggest close monitoring with periodic echocardiography (1,2,15).

### Mood changes and impulse control disorders

Patients with hyperprolactinemia, compared with control individuals, appear to have reduced quality of life, different personality profiles, and increased rates of anxiety and depression. Some reports have associated dopamine agonists with severe depression, manic episodes, and psychosis. Impulse control disorders are characterized by impulsive behaviors that interfere with the individual's daily life and include compulsive buying and eating, pathological gambling, punding (performance of repetitive tasks), and hypersexuality, among other manifestations. The risks and benefits of treatment with dopamine agonists in these circumstances must be assessed together with a psychiatrist to decide on the best course of action, if either maintaining the dopamine agonist at a lower dose with concurrent psychiatric monitoring/treatment or considering neurosurgery in cases involving tumor growth (16,17).

### Rare complications of clinical treatment of prolactinomas

Cerebrospinal fluid fistula, a rare complication, may occur spontaneously or after tumor size reduction in patients with prolactinoma. It usually occurs at the start (weeks to months) of treatment with dopamine agonists and is generally associated with macroprolactinomas invading the sphenoid sinus. In this complication, the tumor is believed to function like a cork initially. Once the tumor tissue undergoes necrosis with treatment, the tumor can no longer block the flow of cerebrospinal fluid, resulting in fluid leakage. Treatment (surgery) must be implemented to correct the fistula and prevent meningitis (18).

Pituitary apoplexy is characterized by an acute infarction and/or hemorrhage within the pituitary gland, leading to an abrupt expansion of tissue volume within the sellar region. These changes may cause a deficiency of pituitary hormones, headache, visual impairment, cranial nerve palsy, and, eventually, impaired consciousness. Prolactinomas are the pituitary tumors most commonly associated with apoplexy, and the risk factors for this complication include the use of dopamine agonists and pregnancy. Pituitary apoplexy is rare, but new or worsening headache during prolactinoma treatment requires urgent imaging of the pituitary to identify the diagnosis. Management of pituitary apoplexy must be carried out by a multidisciplinary team (19).

Rarely, vision deterioration may occur during dopamine agonist treatment despite normalization of prolactin levels. One cause for this complication is herniation of the optic chiasm, which can be diagnosed by pituitary MRI. Dose reduction or interruption of the dopamine agonist and neurosurgery (chiasmopexy) may be indicated after assessment of the patient by a multidisciplinary team (18).

### What are the contraindications to the use of dopamine agonists?

Contraindications to dopamine agonist treatment include breastfeeding, hypersensitivity to ergotamine derivatives, uncontrolled high blood pressure, severe cardiovascular disease, history of untreated coronary disease, and uncontrolled psychiatric disease. In cases of uncontrolled cardiac disease, the discussion about the risks and benefits of dopamine agonist use should include a cardiologist.

### How to define resistance to dopamine agonists?

There is still no universal consensus on the definition of resistance to dopamine agonists or the dose established to identify this complication. Several criteria to define resistance based on prolactin levels have been suggested, including failure to attain normal levels, failure to reduce levels conducive to ovulation, and failure to reduce levels by 50% (1). The degree of reduction in prolactin level required for normal functioning of the gonadal axis varies between individuals. Therefore, a reasonable definition of resistance to dopamine agonists is a failure to achieve normal prolactin levels or symptom control. For macroprolactinomas, resistance to dopamine agonists may be considered when little or no reduction in tumor size is observed. Various criteria have been proposed in this regard, including a reduction of less than 30% in the largest tumor diameter.

About 20%-30% of all prolactinomas are resistant to dopamine agonists. Notably, complete resistance to these agents is rare. Complete resistance to dopamine agonists is characterized by a lack of effect of these agents on prolactin levels. In contrast, partial resistance is defined by a decrease in prolactin level of at least three times the upper limit of normal without achieving complete level normalization (20). Additional response to dopamine agonists may occur over the long term. Not every woman with resistance to these agents requires treatment change. For example, asymptomatic women with a stable adenoma that does not induce any mass effect may not require treatment adjustment.

### What mechanisms and clinical factors are associated with resistance to dopamine agonists?

The main mechanism of resistance to dopamine agonists involves a reduced expression of dopamine D2 receptors, particularly their short isoform. Younger age (*e.g.*, children and adolescents) at diagnosis, male sex, tumoral invasion into cavernous sinuses, and a predominantly cystic pattern in the tumor are the most common factors associated with resistance to dopamine agonists (21). Patients who experience loss of response (secondary resistance) despite adherence to clinical treatment should be referred to a specialized center for investigation of potential biological changes or malignant transformation of the tumor.

### What is the management in cases of dopamine agonist resistance?

**Figure 1:**
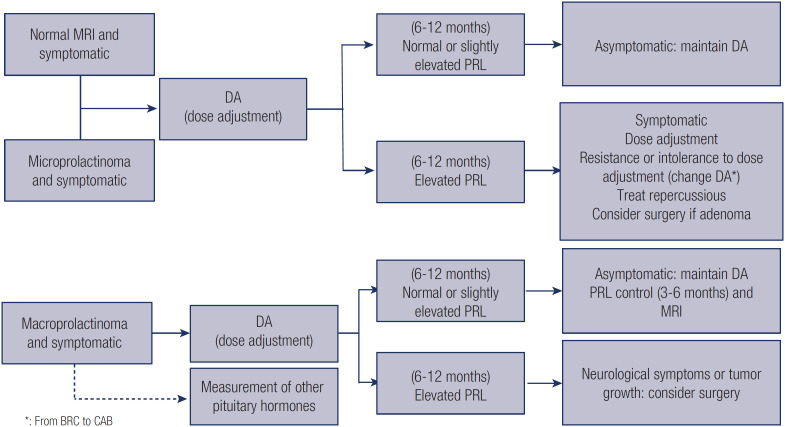
Flow chart of treatment of idiopathic hyperprolactinemia and prolactinoma.

Abbreviations: BRC, bromocriptine; CAB, cabergoline; DA, dopamine agonist; MRI, magnetic resonance imaging; PRL, prolactin.

Patients who develop resistance to bromocriptine may be switched to cabergoline (1,22). In contrast, patients resistant to cabergoline only occasionally respond to bromocriptine (1,22). Patients who tolerate cabergoline well but demonstrate partial resistance to doses up to 2 mg weekly may benefit from a larger dose. However, the patient should be advised about potential long-term side effects with higher doses, particularly the risk of cardiac valve fibrosis. In these cases, it is advisable to attempt a dose reduction once prolactin levels normalize. Notably, little benefit has been shown with cabergoline doses above 3.5 mg weekly, which is the maximum dose and should be used only under close monitoring for side effects (21). Patients experiencing complete or partial resistance to dopamine agonists may benefit from neurosurgical treatment of the prolactinoma, which is usually performed via a transsphenoidal route. The surgery must be performed at a referral center specializing in the treatment of pituitary tumors, with an experienced multidisciplinary team overseeing the process (Figure 1) (23).

### How to manage hyperprolactinemia in women who are infertile or pregnant?

Hyperprolactinemia is characterized by anovulatory cycles, oligomenorrhea or amenorrhea, and luteal phase insufficiency and affects 15%-20% of all infertile women (24). Although bromocriptine is the dopamine agonist most studied in pregnant women, cabergoline appears to be more effective in restoring fertility and has better tolerability. More than 950 pregnancies induced with cabergoline are currently described in the literature, and the reported cases show no increased frequency of spontaneous abortion, premature birth, multiple pregnancies, or neonatal malformations (25). Physical and developmental abnormalities have not been observed in studies that have included approximately 230 children exposed in utero to cabergoline and followed up to the age of 12 years (26). The possibility of transsphenoidal surgery may be discussed with women with prolactinoma who do not experience the return of ovulation and remain with prolactin levels increased despite using cabergoline at maximum tolerable doses. In this case, the patient must be informed about the potential risk of hypopituitarism after surgery and the consequence of this complication on fertility (27). When ovulation is not restored with both treatments (dopamine agonists and transsphenoidal surgery), it may be induced with clomiphene citrate or gonadotropins, ideally after normal prolactin levels are reached (28). Hyperplasia of normal lactotrophs and tumor cells may occur during pregnancy as a result of increased estrogen levels (26,29).

**Figure 2:**
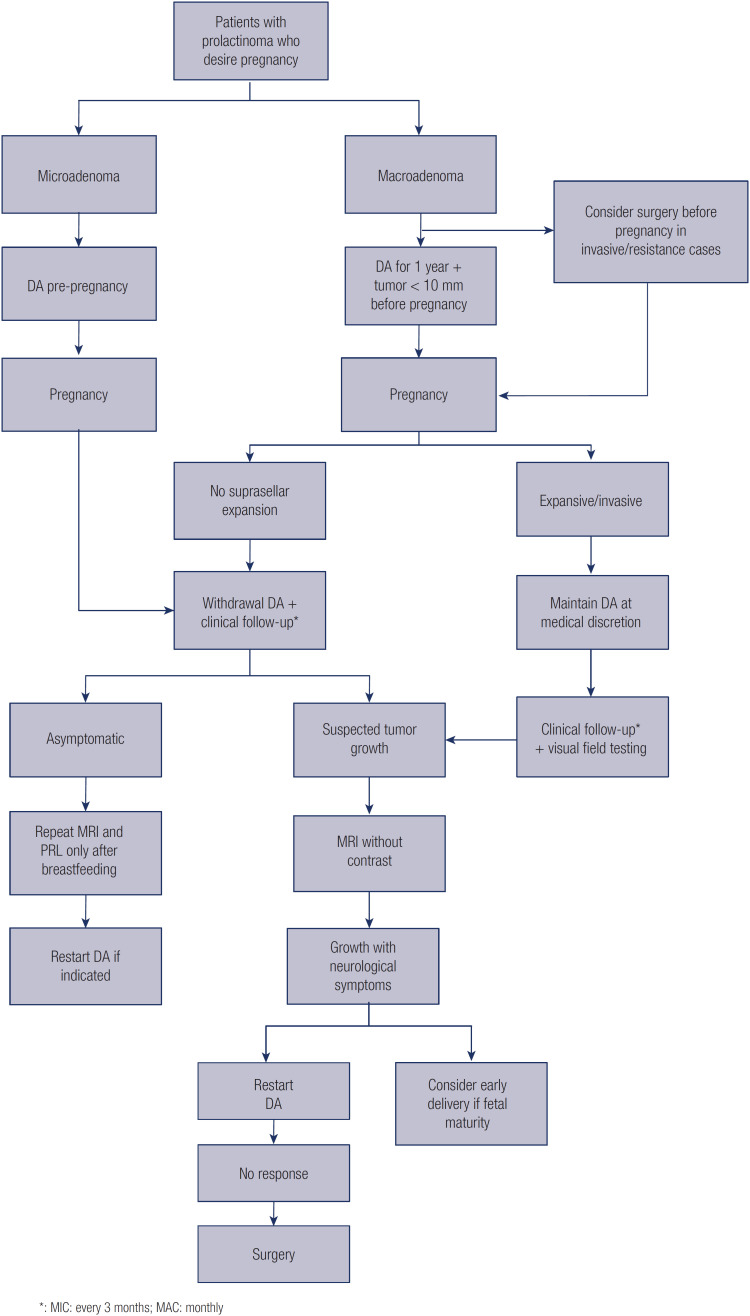
Flow chart of management of prolactinomas during pregnancy.

Abbreviations: DAs, dopamine agonists; PRL, prolactin; MAC, macroadenomas; MIC, microadenomas; MRI, magnetic resonance imaging.

The management of prolactinomas during pregnancy depends on the tumor size. Despite the effects of pregnancy on prolactinomas, the actual risk of growth leading to symptoms (e.g., headache, visual changes) during pregnancy is around 2.5% for microadenomas and 20% for macroprolactinomas without prior surgery or irradiation (26). Furthermore, the risk of apoplexy during pregnancy is considerable in the case of macroprolactinomas (27). Prepregnancy duration of dopamine agonist treatment (particularly for longer than 1 year), small tumors, and lower prolactin levels before pregnancy reduce the risk of tumor growth and increase the chance of tumor regression after pregnancy and lactation. Indeed, a recent Brazilian multicenter study showed that short treatment (<2 years) before pregnancy was associated with significantly higher rates of tumor growth in 194 (233 pregnancies) women with prolactinoma in whom pregnancy was induced with cabergoline (25,30). Considering the low risk of growth of microadenomas during pregnancy, treatment suspension is recommended after pregnancy is confirmed (1,29). In patients with macroadenomas who wish to become pregnant, dopamine agonists are recommended for at least 1 year to reduce tumor dimensions to less than 10 mm. If the tumor reduces in size, discontinuation of the medication during pregnancy may be discussed. In cases with suprasellar expansion or without response to dopamine agonist treatment, transsphenoidal surgery should be considered before pregnancy (26). Pregnant women with operated macroadenomas have a chance of tumor expansion or growth equal to that of women with microadenomas, i.e., around 2.5%. If a woman becomes pregnant after a short period of dopamine agonist use, the tumor size has not decreased, and there has been no previous surgery or radiotherapy, the medication may be maintained after discussion with the patient, particularly if the tumor is close to or abuts the optic pathways (1). In these cases, the medication must be maintained at the same dose used before pregnancy (Figure 2).

Both bromocriptine and cabergoline cross the placental barrier. A European study has found that women exposed to either bromocriptine or cabergoline, compared with those not exposed to these agents, had significantly increased rates of pregnancy loss and prematurity, suggesting an effect related to the medication class and not specifically to cabergoline (31). In a study published in 2020 (25), the miscarriage rate among women who discontinued cabergoline shortly after pregnancy diagnosis was lower (7.5%) than in those who maintained the medication by medical advice or inadvertently (38%). The woman's age at pregnancy, prolactin levels, cabergoline dose, gestational age at cabergoline discontinuation, or concurrent comorbidities were not associated with abortion rates. Despite the potential effect of cabergoline on abortion rates, no associations have been observed between maintaining cabergoline after the first trimester and preterm birth, congenital malformations, or neurodevelopmental changes (25).

Pregnant women who discontinue dopamine agonists are not required to undergo specialized prenatal care. In these patients, signs of tumor growth can be monitored through careful assessment of medical history focused on visual field changes and onset or increased frequency of headaches. It is advisable to conduct these assessments every 3 months for patients with microadenomas and monthly for those with macroadenomas. Neurological evaluation may eventually be performed. In patients with macroadenomas who discontinue the dopamine agonist, quarterly visual field testing should be considered, irrespective of the presence of neurologic symptoms.

Prolactin levels should not be measured during pregnancy, as they increase physiologically up to 10 times compared with prepregnancy levels. Additionally, pituitary imaging should be avoided during pregnancy and is only recommended in cases in which tumor expansion is suspected, as suggested by the onset of frequent headaches or visual field changes reported by the patient or detected on visual field testing. In patients in whom pituitary MRI is required during pregnancy, contrast (*e.g.*, gadolinium) should not be used. In women who develop symptom recurrence and confirmed tumor growth after interruption of dopamine agonists, the medication must be reintroduced (1). In the absence of response to drug treatment, decompression surgery could be considered, preferably during the second trimester. Alternatively, if the pregnancy is more advanced, the option of early delivery may be carefully assessed (26,27).

Prolactin levels normalize 1 week after birth in healthy women who do not breastfeed (32). Although nipple sucking stimulates prolactin pulses, serum prolactin levels during breastfeeding are not usually elevated. Therefore, breastfeeding is not contraindicated in women with prolactinoma, except in those with signs of tumor expansion (26). When lactation must be interrupted, a single dose of cabergoline 1.0 mg (2 tablets) in the immediate postpartum period or, in cases of established lactation, cabergoline 0.25 mg (half a tablet) every 12 hours for 2 days (total dose of 1 mg) lead to lactation suppression in 78%-94% of the cases (33).

### Does monitoring for hyperprolactinemia change in menopause?

Estrogen is a prolactin-releasing factor. The decline in estrogen production during menopause results in a diminished stimulatory effect of this hormone on prolactin secretion and proliferation of lactotrophs. As menstrual cycles cease in menopause, the warning symptoms of hyperprolactinemia become less evident. Although these are physiological changes, evidence of the relationship between the hormonal changes and the production of prolactin during this period is scant (34-36).

### Does menopause change the management of hyperprolactinemia diagnosed during the reproductive period?

Women with microadenoma or idiopathic hyperprolactinemia diagnosed during their reproductive years who transition into menopause and remain asymptomatic may choose to forego dopamine agonist treatment, considering the physiological hypoestrogenism in menopause. On the other hand, those with macroprolactinomas who experience a lesser degree of tumor reduction with dopamine agonists may require continuing medication to avoid compressive effects, depending on the tumor's size and behavior during treatment (35,37).

According to a study, 45% of 11 women with microadenomas experienced remission of hyperprolactinemia after menopause (38). A multicenter study of 29 postmenopausal women diagnosed with prolactinomas during reproductive years (22 with microadenomas and 7 with macroadenomas) found that, after dopamine agonists suspension, 90% of the patients with microadenomas had remission of hyperprolactinemia and tumor reduction and 50% of them had no residual tumor, while in those with macroadenomas, prolactin levels increased slightly above the normal range (39). In another study, including 28 women with microadenomas and macroadenomas who had dopamine agonists suspended after menopause, the prolactin levels increased in 15%, reduced in 33%, and did not normalize or remained normal in 52% of them (40). The tumors grew in 7% of the study participants, and no relationship was observed between remission and duration of treatment, prolactin level, or tumor size at the time of diagnosis or discontinuation of dopamine agonists (40). Thus, women with prolactinomas diagnosed during reproductive years who have optimal prolactin levels and tumors without evidence of compressive effect may suspend dopamine agonists after menopause. Up to two thirds of these women will present remission and 33% will experience recurrence of hyperprolactinemia. Tumor disappearance and stabilization in size have been reported in 58% and 59% of the women, respectively, and tumor growth has been observed in less than 3% of the cases (35,37). Interruption of dopamine agonists with careful monitoring of the tumor may be suggested in selected cases of postmenopausal women with macroprolactinomas who experienced considerable tumor reduction and have no apparent risk of compression of the optic chiasm (37).

After menopause and discontinuation of dopamine agonists, we recommend follow-up with clinical evaluation and measurement of prolactin levels every 6 months in the first year and pituitary MRI at 12 months or earlier if symptoms of tumor mass effect develop. Treatment with dopamine agonists should be maintained in cases of expansive and/or invasive tumors.

### What are the unique characteristics of prolactinomas diagnosed after menopause?

The true incidence of prolactinomas diagnosed after menopause is unknown. In general, the diagnosis of prolactinomas at this stage is established in the presence of neurologic symptoms; macroadenomas are more frequent in this situation, with serum prolactin levels generally high, and an optimal response to dopamine agonists is obtained even with late diagnosis (41).

### What is the management of hyperprolactinemia secondary to medications?

Antipsychotics are the most relevant of all medications causing hyperprolactinemia, as they increase prolactin levels more frequently and at greater intensity than other drugs. This type of medication must be managed by a psychiatrist, and a general practitioner or physician of another specialty should not suspend them without prior contact with the prescriber. Hyperprolactinemia secondary to antipsychotics should be treated when associated with hypogonadotropic hypogonadism due to the effects of hypoestrogenism. The following three management alternatives are possible in this situation: (1) change the medication that induced the hyperprolactinemia, (2) initiate hormone replacement therapy with estrogen, which should be associated with progestogen in patients with uterus, and (3) start a dopamine agonist (in this case, close monitoring is required, since the dopamine agonist and the antipsychotic may have opposing effects that increase the risk of exacerbation of the psychotic condition) (42). A recent literature review has shown that patients with hyperprolactinemia induced by antipsychotic medications may benefit from treatment with aripiprazole as a replacement or associated medication, since this antipsychotic does not cause hyperprolactinemia (42).

## FINAL CONSIDERATIONS

This position statement was prepared jointly by the Department of Neuroendocrinology of the Brazilian Society of Endocrinology and Metabolism (SBEM) and the National Specialized Commission on Gynecological Endocrinology of the Brazilian Federation of Gynecology and Obstetrics Associations (Febrasgo). The aim of this document is to provide updated information to assist gynecologists, endocrinologists, and primary care physicians in treating patients with hyperprolactinemia.
